# Structural Specificity of Polymorphic Forms of α-Synuclein Amyloid

**DOI:** 10.3390/biomedicines11051324

**Published:** 2023-04-29

**Authors:** Irena Roterman, Katarzyna Stapor, Leszek Konieczny

**Affiliations:** 1 Department of Bioinformatics and Telemedicine, Jagiellonian University—Medical College, Medyczna 7, 30-688 Krakow, Poland; 2 Department of Applied Informatics, Silesian University of Technology, Akademicka 2A, 44-100 Gliwice, Poland; 3 Medical Biochemistry, Jagiellonian University—Medical College, Kopernika 7, 31-034 Krakow, Poland

**Keywords:** α-synuclein, amyloid, misfolding, external force field, hydrophobic core

## Abstract

The structural transformation producing amyloids is a phenomenon that sheds new light on the protein folding problem. The analysis of the polymorphic structures of the α-synuclein amyloid available in the PDB database allows analysis of the amyloid-oriented structural transformation itself, but also the protein folding process as such. The polymorphic amyloid structures of α-synuclein analyzed employing the hydrophobicity distribution (fuzzy oil drop model) reveal a differentiation with a dominant distribution consistent with the micelle-like system (hydrophobic core with polar shell). This type of ordering of the hydrophobicity distribution covers the entire spectrum from the example with all three structural units (single chain, proto-fibril, super-fibril) exhibiting micelle-like form, through gradually emerging examples of local disorder, to structures with an extremely different structuring pattern. The water environment directing protein structures towards the generation of ribbon micelle-like structures (concentration of hydrophobic residues in the center of the molecule forming a hydrophobic core with the exposure of polar residues on the surface) also plays a role in the amyloid forms of α-synuclein. The polymorphic forms of α-synuclein reveal local structural differentiation with a common tendency to accept the micelle-like structuralization in certain common fragments of the polypeptide chain of this protein.

## 1. Introduction

The phenomenon of misfolding α-synuclein (A-Syn) is of particular importance in the context of pathologies such as numerous neurodegenerative diseases [1,2,3,4,5,6]. Mutational changes in A-Syn are identified in synucleinopathies [7]. However, some mutations have been also observed to prevent amyloid transformation [8]. From the point of view of the structuring process itself, the participation of intrinsically disordered proteins is also considered [9]. An interesting observation concerns a certain synergy in the construction of A-Syn fibrils in the presence of amyloids from the Aβ (1–42) group [10]. The phenomenon of amyloid A-Syn formation turns out to be related to the transition liquid-to-solid phase observed in the liquid–liquid phase separation, leading to the formation of this protein’s amyloids [11]. A chain segment was also identified—NAC (71–82), which influences axonal accumulations and the binding of A-Syn to membranes [12].

A review of the above-mentioned studies suggests a significant dependence of the structuring of A-Syn amyloid fibrils on external factors. In the present work, an analysis of the assessment of the influence of such factors was carried out based on the fuzzy oil drop model, which (in a modified form) takes into account the influence of factors other than water influencing the characteristics of the external force field participating in protein structuring, in particular, the formation of fibrils [13,14,15].

The main idea of the presented model is to express the specificity of the external force field participating in the folding process and thus influencing the protein folding. The main environment in the form of polar water supports the formation of the polypeptide chain in micelle-like construction (hydrophobic core with polar shell). The structures of different proteins can be analyzed using the micelle-like hydrophobicity distribution as the reference distribution. The form and degree of the discrepancy in respect to this reference is examined as carrying the specific information related to structural specificity including the biological activity. The model description in its short form is aimed at a proper interpretation of the presented results. 

Hydrophobic core with polar surface can be modeled by 3D Gauss function spread over the protein molecule. The values of this function in a particular point (the position of effective atoms representing residue) represent the theoretical level of hydrophobicity in that point. However the hydrophobicity distribution observed in protein is the effect of inter-residual hydrophobic interactions depending on the intrinsic hydrophobicity of each residue and the distance between them. The differences between the observed and the theoretical values express the similarity/dissimilarity of hydrophobicity distribution in protein with respect to an idealized/theoretical one. It can be assessed quantitatively by parameter Relative Distance (RD) expressing the distance between idealized and observed distribution of hydrophobicity in a protein molecule. However, water directing the folding process toward micelle-like forms is not the only one environment influencing the structure of a protein. The membrane environment expects the exposure of hydrophobicity and (in case of trans-membrane channels) the central part of protein represents a low level of hydrophobicity. This is why the complementary function to 3D Gauss is proposed to describe the hydrophobicity distribution in membrane proteins. The level to which the complementary 3D Gauss function modifies the micelle-like distribution assesses the influence of other than water environmental conditions. A detailed description of the methodology is given in Materials and Methods. 

## 2. Materials and Methods

### 2.1. Data 

Polymorphic structures of A-Syn as available in PDB [16] (accessed December 2022) are the objects of analysis. The list of proteins with their short characteristics (chain fragment) is given in Table 1. 

### 2.2. Fuzzy Oil Drop Model Description

The model called fuzzy oil drop assumes the distribution of hydrophobicity in a protein molecule in a form corresponding to that in micelles. The aqueous environment directs bipolar molecules to form micellar structures with hydrophobic portions in the central part of the protein separated from the aqueous environment by a polar layer. Amino acids are a set of 20 bipolar molecules with different degrees of the relationship between polar groups and hydrophobic parts. Such molecules avoid contact with water by targeting the central part of the molecule while exposing polar residues on the surface. Amino acids as bipolar molecules undergo a similar process of micellization. The restriction of the freedom of movement of individual amino acids (peptide bonds imposing a specific neighborhood) results in an incomplete possibility of micelle-like ordering. Quantification of the degree of micelle-like structuring in relation to areas showing deviations from the micelle-like system is the main idea of fuzzy oil drop model (FOD). To describe such a structure, a 3D Gaussian function was used, the values of which at certain points of the protein show the so-called theoretical level of idealized hydrophobicity—$T_{i}$. 3D Gaussian function spanned over the protein body with dimensions depending on the shape of the molecule (different values of *sigmaX*, *sigmaY* and *sigmaZ*).

(1) $$H_{i}^{T}=\frac{1}{H_{sum}^{T}}\exp\left(\frac{-{\left(x_{i}-\bar{x}\right)}^{2}}{2\sigma_{x}^{2}}\right)\exp\left(\frac{-{\left(y_{i}-\bar{y}\right)}^{2}}{2\sigma_{y}^{2}}\right)\exp\left(\frac{-{\left(z_{i}-\bar{z}\right)}^{2}}{2\sigma_{z}^{2}}\right).$$

The actual level in points representing effective amino acids (average position of atoms of a given amino acid) is the result of hydrophobic interactions depending on the intrinsic hydrophobicity of each amino acid and the distance between interacting residues. The function proposed by Levitt [34] was used to determine the level of the observed level of hydrophobicity—Oi.

(2) $$H_{i}^{O}=\frac{1}{H_{sum}^{O}}\sum_{j}\left\{\begin{array}{l} \left(H_{i}^{r}+H_{j}^{r}\right)\left(1-\frac{1}{2}\left(7{\left(\frac{r_{ij}}{c}\right)}^{2}-9{\left(\frac{r_{ij}}{c}\right)}^{4}+5{\left(\frac{r_{ij}}{c}\right)}^{6}-{\left(\frac{r_{ij}}{c}\right)}^{8}\right)\right)\;for\;r_{ij}\leq c\\ 0,\;\;for\;r_{ij}>c \end{array}\right..$$

Comparison of these two distributions requires normalization of both of them (appropriate *H_sum_* in Equations (1) and (2)).

The degree of similarity of the *T* (theoretical) and *O* distribution (observed) is determined using the Kullback–Leibler divergence entropy function [35] $D_{KL}$ for the O|T relation:

(3) $$D_{KL}\left(P|Q\right)=\sum_{i=1}^{N}P_{i}\log_{2}\frac{P_{i}}{Q_{i}}.$$

where $P_{i}$—analyzed distribution, in our model: *O*; $Q_{i}$—reference distribution, in our model: *T* or *R* (defined below). 

The value determined with this function cannot be interpreted directly. Therefore, measures of the degree of similarity of the *O* distribution to the uniform *R* distribution are introduced—where $R_{i}=1/N$, where *N* is the number of amino acids in the chain. The *R* distribution represents the status of hydrophobic core absent in contrast to the 3D Gauss function. The *R* distribution assumes a uniform hydrophobicity distribution without any variation in the level of hydrophobicity at any point in the protein. Comparing the values of $D_{KL}$ for the relation O|T to $D_{KL}$ for O|R makes it possible to conclude that the *O* distribution is similar to one of the reference distributions. To eliminate the use of two values for the description of one object, the *RD* parameter was introduced, defined as follows:

(4) $$RD=\frac{D_{KL}(O|T)}{D_{KL}\left(O|T\right)+D_{KL}(O|R)}.$$

An *RD* value less than 0.5 indicates the presence of a hydrophobic core while *RD* > 0.5 indicates the uniform dispersion of hydrophobicity throughout the protein body.

Proteins with hydrophobic distribution consistent with the 3D Gaussian function have been identified: down-hill, fast-folding, ultra-fast-folding, and antifreeze type II proteins [36,37]. Additionally, it should be noted that the vast majority of domains treated as independent structural units represent 3D Gaussian function distribution [38].

Water is not the only environment for proteins to be active. A large group of proteins is active in the membrane environment, i.e., in a highly hydrophobic environment. The protein stabilized in the membrane exposes hydrophobic residues on the surface, and in the case of channels, in the central part, the level of hydrophobicity is the lowest. This means the inverse distribution to the 3D Gaussian function is in the form (1-3D Gauss). It turns out, however, that the hydrophobicity distribution in membrane proteins shows a hydrophobicity distribution consistent with the 3D Gaussian distribution modified only by the distribution (1-3D Gauss). The final form of the force field for membrane proteins takes the form:

(5) $$M_{i}={\left[3DG+K{\left(1-3DG\right)}_{n}\right]}_{n}.$$

(6) $$M_{i}={\left[T_{i}+K{\left(T_{MAX}-T_{i}\right)}_{n}\right]}_{n}\;$$

where the index *n* denotes the normalization of a given distribution.

The field description in the form of $M_{i}$ is the essence of the model called FOD-M (M—modified). The most important parameter in the definition of this field is the *K* parameter, which indicates to what extent the standard field based on 3D Gauss function is modified by other factors, including hydrophobic ones in particular, and how it affects the folding of a given protein. The folding protein reproduces the mechanism imposed on it by the surrounding force field. Therefore, the interpretation of the *K* parameter value determines the specificity of the surrounding field and the strength of its influence on the process of structuring the polypeptide chain. The value of *K* is determined for the minimum value of $D_{KL}$ for the relation (O|M).

**Figure 1:**
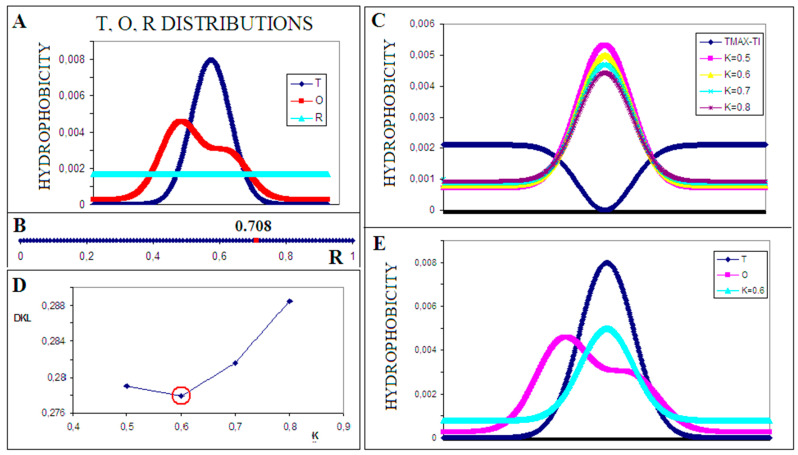
Visualization of the FOD-M procedure using the example limited to 1DGauss function. (**A**) distributions: T, O, and R put together. (**B**) the R value calculated for the example in (**A**). (**C**) M distributions for different K values to search the optimal K value for O distribution. The dark blue line—the (T_max_ − T_i_) distribution (**D**), the optimal K value appears as K = 0.6 for the given example (minimal D_KL_ value for K = 0.6). (**E**) distributions *T*, *O*, and *M* for K = 0.6 for the given example.

The visualization of the FOD-M model given in Figure 1 is aimed to facilitate the interpretation of results obtained on the basis of the FOD-M model. 

The FOD-M model has been used to describe the structures of membrane proteins [13,14,15] and the structures of amyloids [39,40].

From the *T*, *O*, and *M* profiles determined for complete chains, it is possible for step-wise elimination of successive residues or specific segments in order to identify the significance of a given segment. If, after elimination, the value of *RD* decreases, this means that the given eliminated segment is a carrier of a deliberate disturbance of the micelle-like degradation. The system of ideal micelle-like distribution would not show any biological activity. The eliminated residues carrying significant differences in $O_{i}$ to $T_{i}$ very often turn out to be the residues responsible for biological activity (e.g., catalytic residues in enzymes). The elimination of residues with high differences, leading to an *RD* value of less than 0.5, allows the identification of the part of the protein which, built according to the micelle rules, is responsible for the solubility in the aqueous medium. Any residual (or segment) elimination requires normalization of the distribution. 

In the Results section, the values of *RD* and *K* parameters for the discussed amyloid forms of A-Syn are given. The values of these parameters are determined for the structural units: single chain, proto-fibril, and super-fibril. The residues causing the increase in *RD* and *K* values were also identified. This operation is aimed at demonstrating the presence of amyloid parts in fibrils that may be the effect of the influence of the aquatic environment.

The values of *RD* for the analyzed proteins determine the degree of restoration of the micelle-like system, while the value of the *K* parameter determines the degree of differentiation of the environment influencing the structuring of a given protein.

In order to organize the description of the FOD-M model, its parameters should be defined:

*T*—hydrophobicity distribution resulting from the assumption of an idealized hydrophobicity distribution consistent with the micelle-like system (3D Gaussian function spread over the body of a given protein), the generation of which is supported by a polar water environment;

*O*—distribution actually present in the protein as a result of inter-amino acid interactions (depending on the distance between the effective atoms representing a given amino acid and on the intrinsic hydrophobicity of each amino acid);

$D_{KL}$—entropy divergence value measuring the degree of differences between the *O* distribution against T ($D_{KL}$ for the (O|T) relationship) and between the *O* distribution and the *R* distribution (reference distribution without the presence of a hydrophobic core—$D_{KL}$ for the (O|R) relationship) (Figure 1A); 

*RD*—the value of this parameter determines how close the *O* distribution is to the *T* distribution (0 < *RD* < 1). An *RD* value of <0.5 indicates the presence of a centric hydrophobic core (Figure 1B);

*M—*distribution modified by environment identified by gradual addition (parameter K) of the opposite to micelle-like distribution (T_MAX_-Ti); the K value defined by minimal *D_KL_* for (O|M) distance (Figure 1C);

*K*—the value of this parameter determines the degree of modification of the external force field in relation to the aquatic environment. The higher the value of this parameter, the more distant is the characteristic of the external force field to the polar water environment (then *K* = 0). The value of *K* is determined by looking for the distribution of *M* with the smallest $D_{KL}$ value for the relation *O* to *M*—it is a modification of the field which, influencing the folding process, led to the distribution determined by the size of the *RD* parameter (Figure 1D,E).

### 2.3. Programs Used

The potential used has two possible access to the program:

The program allowing calculation of *RD* as well as T and O distributions is accessible upon request on the CodeOcean platform: https://codeocean.com/capsule/3084411/tree accessed 14 January 2023. Please contact the corresponding author to obtain access to your private program instance.

The application—implemented in collaboration with the Sano Center for Computational Medicine (https://sano.science accessed 14 January 2023) and running on resources contributed by ACC Cyfronet AGH (https://www.cyfronet.pl accessed 14 January 2023) in the framework of the PL-Grid Infrastructure (https://plgrid.pl accessed 14 January 2023)—provides a web wrapper for the above-mentioned computational component and is freely available at https://hphob.sano.science (accessed 14 January 2023).

The VMD program was used to present the 3D structures [41].

## 3. Results

The presented results consist of two parts. The first one represents the analysis of reference proteins to facilitate the interpretation of the results concerning A-Syn. The first part is aimed to show proteins of different status in respect to the FOD model.

1. Analysis of exemplary structures of different proteins:
  1.1. globular one (distribution O close to T distribution, K = 0.0),
  1.2. protein acting in periplasmic environment (hydrophobic core absent = K = 0.7)
  1.3. membrane domain of transmembrane protein playing the role of channel—hydrophobicity distribution opposite to micelle-like—exposure of hydrophobicity on the surface.
2. Analysis of the amyloid forms of A-Syn structures available in PDB [16].

The proteins discussed in point 1 are presented as an introduction to the A-Syn analysis to make possible the comparable analysis of amyloid structures versus the proteins of WT structure acting in different environments. 

### 3.1. Exemplary Proteins of Different Status in the FOD-M Model Assessment

The basic assumption of the FOD model is to treat amino acids as bipolar molecules with a variable polar to hydrophobic ratio. On this basis, it was assumed that protein folding in an aqueous environment relies on such a chain arrangement that the micelle-like arrangement is reproduced as much as possible. The degree of similarity of the hydrophobicity distribution to the micelle system is varied. In the present introductory section, examples of proteins with a gradually increasing maladjustment to an idealized system expressed by a distribution called T (consistent with the 3D Gaussian distribution) are presented.

#### 3.1.1. Proteins with a High Degree of Micelle-like Order

Proteins with a highly ordered hydrophobicity distribution, reproducing a micelle-like system: centrally located hydrophobic core with a polar surface—these are proteins from the down-hill, fast-folding, ultra-fast-folding group [37]. In addition, it was found that the domains in multi-domain proteins also represent this type of arrangement [38]. Here is an example of the protein albumin binding domain from *Finegoldia magna* (PDB ID—1PRB [42], a protein listed in the fast-folding group [37]).

**Figure 2:**
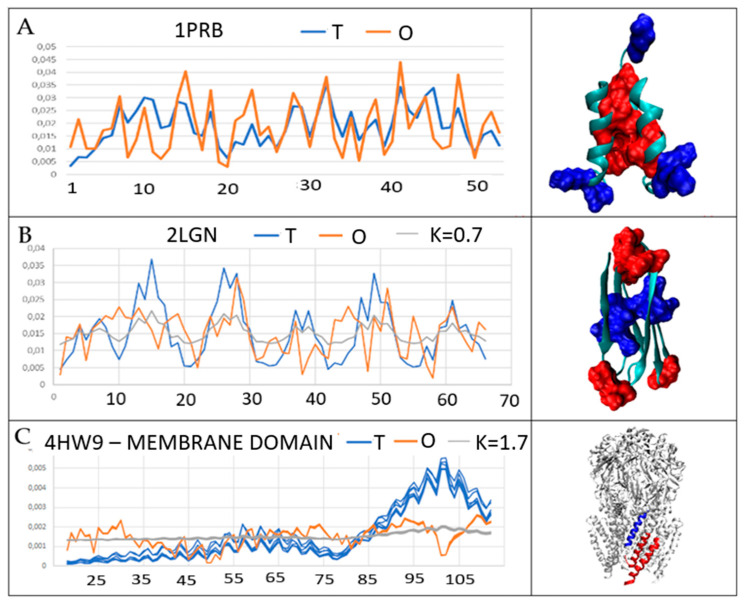
Comparison of *T*, *O*, and *M* profiles for sample proteins: (**A**) protein belonging to the fast-folding group showing structuring (hydrophobicity distribution) consistent with the micelle-like system; the following elements were distinguished in the 3D presentation: the residues of the hydrophobic core—red; surface proteins with hydrophobicity close to zero—blue. (**B**) protein operating in a periplasmatic environment showing the status of *K* = 0.7, which means the share of factors different from polar water. The following were distinguished in the 3D presentation: residues showing local excess of hydrophobicity—red; local deficit of hydrophobicity—blue. (**C**) a protein that represents the cell membrane environment. The following were distinguished in the 3D presentation: residues showing local excess of hydrophobicity—red; local deficit of hydrophobicity—blue. All 7 domains presented on profiles of T and O.

The parameters describing the status of this protein using the FOD model are: *RD* = 0.401 and *K* = 0. The set of appropriate *T* and *O* profiles (Figure 2A) visualizes a high degree of similarity of distribution for the protein in question.

The determined value of the parameter K = 0 allows to conclude that the aquatic environment directed the mutual arrangement of amino acids, aiming at generating a centric core and a polar surface. This results in the solubility of the protein and its low (zero) involvement in interaction with other molecules or proteins.

#### 3.1.2. Protein Folded in the Periplasmic Environment

An example of a protein whose status is expressed by the value of the parameter *RD* = 0.629 and *K* = 0.6 is *Lactococcin 972* antimicrobial protein (PDB ID—2LGN [43]).

The comparison of the *T*, *O*, and *M* profiles reveals the need to modify the form of the external force field that directs the folding process towards a structure deviating from the micelle-like form. The folding of this protein with the help of specialized tools for predicting the structure of proteins provides a form with a micelle-like structure. This proves that the parameterization present in the package is directed at the micellization process without taking into account the different environment, in this case, the environment of the periplasmic space [44].

The comparison of the profiles (Figure 2B) reveals the reconstruction of the specificity of the external force field in the protein structure, bringing the *O* distribution closer to the *M* distribution, i.e., the distribution different from the aquatic environment through the parameter *K* = 0.7 expressing the degree of dissimilarity of this environment from the aquatic environment.

#### 3.1.3. The Influence of the Membrane Environment Expressed by the Value of the K Parameter

Another reference example for the analysis of the A-Syn amyloid forms discussed in this paper is the example of a membrane domain of the transmembrane protein playing the role of channel. Its expected hydrophobicity distribution is radically different to that induced by polar water. Trans-membrane protein—mechanosensitive channel mscs (PDB ID—4HW9 [45]) from *Helicobacter pylori* and its membrane anchored domain in particular shows a distribution that meets the conditions required for stabilization in the hydrophobic environment of the membrane. Here, exposure of hydrophobic residues to the surface is expected, making the effect of the distribution $T_{MAX}-T_{i}$ significant. The value of the parameter *K* = 1.7 for this domain indicates the specificity of the external force field (membrane) as significantly modifying the hydrophobicity order in this protein. Additionally, the significant hydrophobicity deficiency is related to channel construction. High hydrophobicity is expected in the central part, while the empty space (or significantly lower packing) is present in the channel area. 

Comparison of profiles (Figure 2C): *T*—idealized micelle-like distribution (the effect of ordering induced by the polar water environment) and *O*—distribution present in the discussed protein (the result of inter-amino acid interactions) reveals essential differences between these distributions. This means that the structure is the result of an interference with an environment other than polar water in the folding process. The difference between T and O distribution is expressed by RD = 0.834 and external force field participation is expressed by K = 1.7. The 3D presentation of the structure shows a significant exposure of hydrophobic residues on the protein surface needed for the stabilization of the transmembrane domain of mechanosensitive channel (mscs).

The color-highlighted amino acids were identified in the profiles as representing a significant excess (red) and a significant deficit (blue) of hydrophobicity.

The compilation of the discussed three exemplary proteins is aimed at demonstrating the possibility of the FOD-M model in the field of quantitative evaluation of the influence of non-aquatic factors on the hydrophobicity distribution. The values of the K parameter for the examples given express the form of the external force field, which, by participating in the folding process, directs this process towards the generation of a structure with an appropriately changed structuring—often far from micelle-like structuring.

The analysis of the proteins presented here provides an opportunity for a comparative analysis to describe the structures of A-Syn amyloid forms. The given analysis also describes the application of the FOD-M model for the quantitative assessment of the contribution of the altered external force field constituting the environment for the protein folding process.

### 3.2. Analysis of A-Syn Amyloid Forms

As described earlier, the FOD-M model (M-modified—taking into account the participation of environmental factors other than water in shaping the protein structure) was used to describe the set of A-Syn amyloid forms. Each structure is described by *RD* and *K* parameters for the individual chain, proto-fibril, and super-fibril. The combination of these parameters allows determining the process of obtaining structure by the amyloids in question.

The procedure of eliminating the residues disturbing the theoretical *O* distribution was also carried out. In this way, a part of the proto-fibril or super-fibril chain with the order of the micelle-like system was identified. This part of the system is responsible for the enthalpy favorable contact with the surrounding polar water.

Based on the set of these parameters, it is possible to propose a mechanism for the formation of a single-chain structure and proto-fibrils, as well as a mechanism leading to the formation of super-fibrillary systems as an effect of obtaining micelle-like forms to a degree depending on the characteristics of the environment.

The present work is a continuation of the topic presented in [15], where a hypothesis was put forward regarding two scenarios of amyloid transformation. One of them—an example of which is A-Syn—consists in the transition from a far micelle-like state in the native form (high value of K) to the form determined by water conditions, i.e., with a high concentration of hydrophobicity in a micelle-like form with low value of *K*.

The high value of *K* for the native form is the result of the strong influence of axon terminals of presynaptic neurons with which A-Syn is complexed. When freed from this factor that stabilizes the structure far from the globular, A-Syn adapts to the aquatic environment with a tendency to generate a system that meets the favorable entropy–enthalpy conditions in relation to the aquatic environment.

The second scenario proposed in [15], observed in the case of amyloid formed by transthyretin or the light chain of IgG, consists in changing the globular structure, which these proteins represent in their native form (low value of K), into an amyloid structure with a high value of K. This process takes place under the influence of external factors that direct the structuring in the form of fibrils.

This factor can be, for example, the appearance of a high content of the air–water interphase (in the shaking process). This change in water structuring results in a different than natural water effect on the structuring of these proteins.

The current work analyzing numerous structures of A-Syn available in PDB justifies the assumption about the nature of the amyloid transformation process related to the decrease in the value of the parameter *K* expressing the influence of the environment on protein structuring.

The collective summary of the obtained results indicates a significant diversity of structures in the assessment of the fuzzy oil drop model (Table 2).

Determining the status of the above-mentioned structural units requires spreading the 3D Gaussian function on a given unit. Adjusting the shape of the 3D Gauss function consists in selecting the values of *sigmaX*, *sigmaY*, and *sigmaZ* reflecting the shape of the structure of the object.

The amyloid available in PDB ID 2N0A has been taken as a reference to all the others due to the complete chain presence, although only the central part (38–100) shows amyloid structure. The N- (1–37) and C-terminal (101–140) segments show highly disordered forms of the Random Coil type. Therefore, no chain can be considered representative. A high value of *RD* and *K* for a single chain (chain B) cannot therefore be treated as representing the remaining chains of this system.

The list of *RD* and *K* parameters has been ordered according to the increasing value of *RD* of a single chain (Table 2).

This comparison shows a large spectrum of structures from the point of view of their evaluation by means of the hydrophobicity distribution—the *RD* parameter. Examples of micelle-like chain structures are quite numerous. Noteworthy are those with the lowest *RD* value and those with very high *RD* values, indicating a significantly distant system from the system with a centric concentration of hydrophobic residues.

**Figure 3:**
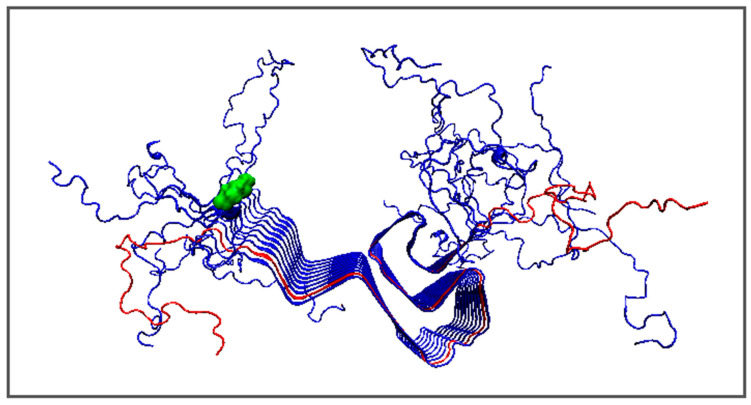
Structure 1–140—amyloid A-Syn. N-terminal residue—green for better navigation. Chain B was distinguished as red.

The analysis of the amyloid structures of A-Syn begins with the characterization of the complete chain amyloid of this protein (PDB ID 2N0A) (Figure 3).

This amyloid is characterized by a fibrillar arrangement only for the central part (40–100) in a chain length of 140 aa. The N- (1–30) and C-terminal (101–140) fragments show a significantly high degree of disorder understood as Random Coil. Therefore, the choice of any chain cannot be representative of a complete polypeptide chain fibril. Distinguished as red in Figure 3, the chain B for which the values of *RD* and *K* are given is clearly not representative of the entire structure of this system.

**Figure 4:**
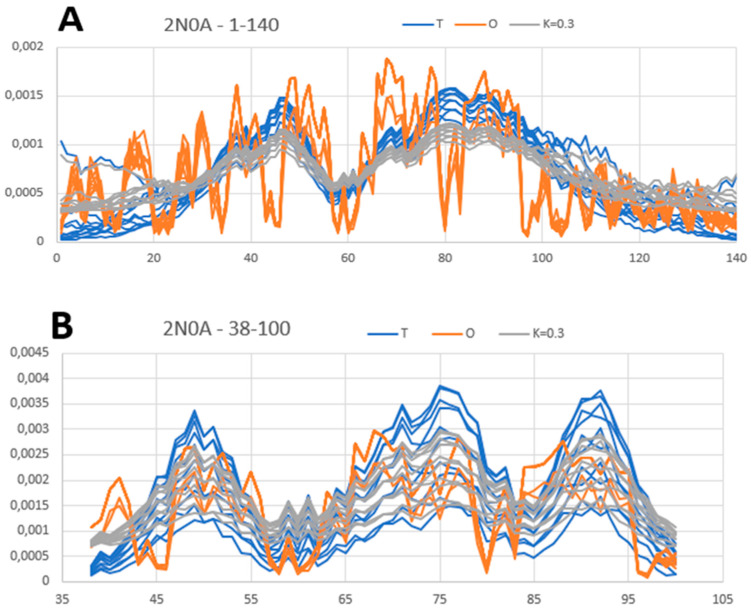
Summary of *T*, *O*, and *M* profiles for A-Syn in the form: (**A**) amyloid including complete chain lengths and (**B**) amyloid part.

The entire structure consisting of 10 chains shows an order of hydrophobicity consistent with a micelle-like distribution with a centrally located hydrophobic core and zones with decreasing levels of hydrophobicity (Figure 4A). This part consists of the disordered structure of the N- and C-terminal sections. The central fibrillar part shows an arrangement consistent with the fuzzy oil drop model (profile—Figure 4B).

The analysis of the profiles for the complete chain reveals some peculiarities. The status consistent with the micelle-like system was achieved despite the presence of single residues showing a significant deviation in the *O* distribution from the *T* distribution. These are the positions: 53 Lys, 45 Lys, 46 Glu, 80 Lys, 83 Glu, 96 Lys, and 97 Lys, which show a significant local hydrophobicity deficit and a 66–71, where there is a local excess of hydrophobicity resulting from the presence of as many as three Vals in this short section. Here, however, it is an excess in the area close to the hydrophobic core. Therefore, its significance is not particularly important for the interpretation of the micelle-like structure. If the excess concerned the exposed part (on the surface), such an excess would be of significant importance.

The analysis of the profiles determined for the fibrillar part places the mentioned disrupting micelle-like system for the entire chain in positions much less disturbing the *T* system.

Despite these position mismatches, A-Syn’s amyloid status is similar to the micelle-like system. Later in this work, while discussing the remaining amyloid structures of A-Syn, attention will be paid mainly to those positions that show maladjustment to the micelle-like status.

In the spectrum of different assessments of micelle-like order in the amyloid forms of A-Syn, a special place is occupied by the form with PDB ID 6CU8. This amyloid is made up of the 43–83 chain segments and thus is part of the fibrillar part of the complete chain. This amyloid in all the forms considered: single chain, proto-fibril, and super-fibril shows an ordering consistent with the *T* distribution.

**Figure 5:**
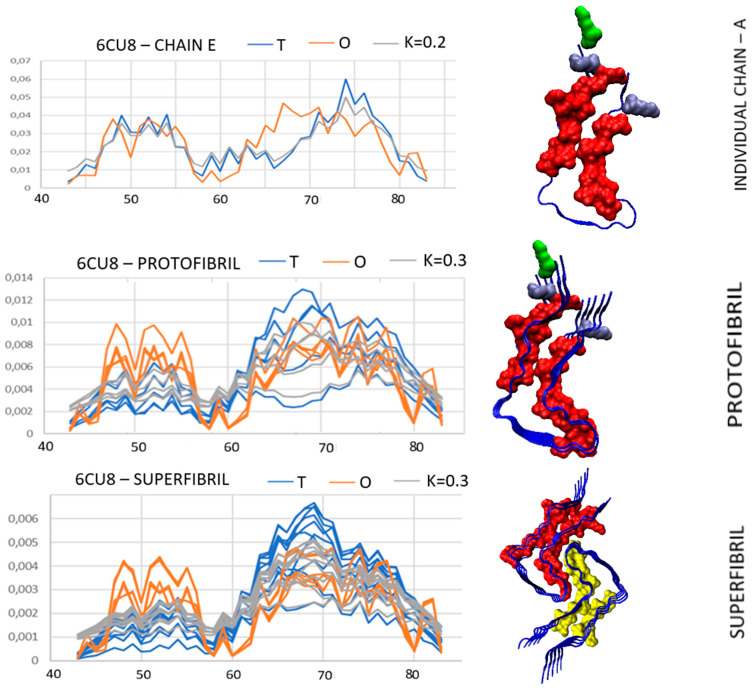
Set of *T*, *O*, and *M* profiles for amyloid structural units with PDB ID 6CU8. Upper—single chain; central—proto-fibril; bottom—super-fibril. Residues distinguished as red—components of the hydrophobic core; yellow segment—component of the hydrophobic core derived from the second proto-fibril. The residues are distinguished as ice-blue—lysine positions: 43, 45, 80 causing a clear deficit in the case of the amyloid structure with PDB ID 2N0A. Green—N-terminal position in these strings.

The micelle-like status of all structural units is the result of the cooperation of polypeptide chains with the water environment (Figure 5). The influence of the external polar force field on the shaping of all the considered structural units is clearly visible here. The polypeptide chain reacted to the environment by creating a micelle-like structure.

The status of the residues introducing a mismatch between the *O* distribution and the *T* distribution in the case of the complete chain turns out to be highly consistent with expectations. The residues L43, L45, L80, and E83 are located on the surface and their high polarity fits exactly with the expectations of the *T* distribution (Figure 5).

The 3D structure presentations reveal an almost perfect hydrophobic core structure in all the structural units discussed here.

The super-fibril structure shows a very good fit of the two proto-fibrils, in which the fit of the structure of the common hydrophobic core seems to be dominant.

**Figure 6:**
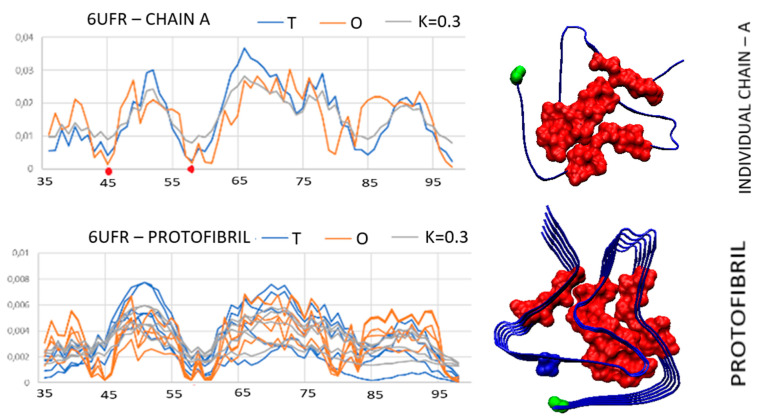

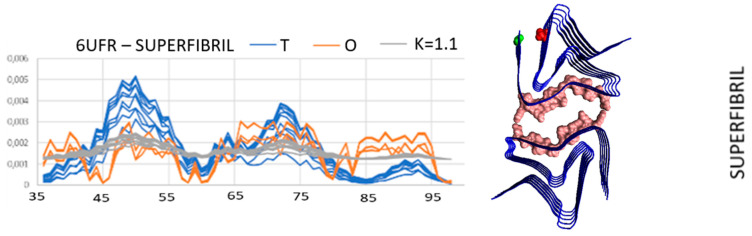
List of *T*, *O*, and *M* 6UFR profiles for: upper—single chain; central—proto-fibril; bottom—super-fibril. Items K45 and E57 highlighted on the profile of a single chain—items responsible for the contact between proto-fibrils. Residues marked pink on the 3D structure—expected hydrophobic core in a super-fibril not observed in the actual structure of this super-fibril.

An example of an amyloid with code PDB ID 6UFR shows a micelle-like arrangement with a centrally located core and a polar surface for a single chain as well as for proto-fibril. The *T* and *O* profiles for a single chain and proto-fibril locate all the residues identified earlier in the case of a complete chain in the appropriate positions in accordance with the *T* distribution (Figure 6). Super-fibril, on the other hand, shows an extremely high maladjustment to the micelle-like system. Instead of a hydrophobic concentration, there is free space at the center of the super-fibril. The interaction between the proto-fibrils is present on the basis of the contact between the two residues E57 and K45 from each proto-fibril. This interaction is clearly polar in nature. The creation of a super-fibril structure cannot be the result of the influence of the aquatic environment, where the intervention of water probably would not lead to the formation of a stable system based on several interactions based on polar interactions.

The discussed amyloid is an E46K mutant observed in cases of hereditary Parkinsonism and Lewy body dementia [19]. According to reports based on experimental research, it is postulated that the current mutation facilitates the formation of the amyloid structure and directs the structuralization of A-Syn by optimizing internal energy. The role of mutations is postulated as a factor facilitating the creation of misfolded forms [19].

**Figure 7:**
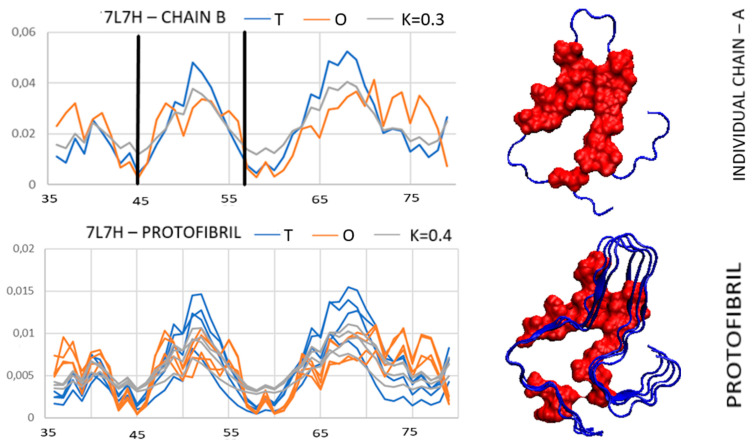

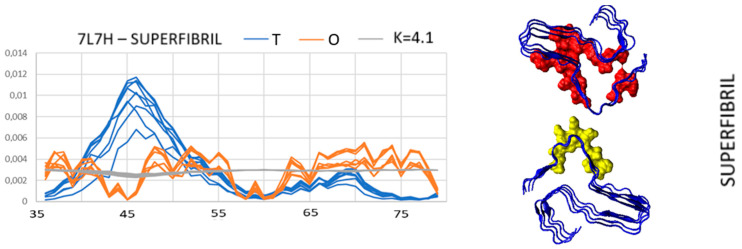
The *T*, *O*, and *M* 7L7H profiles for: upper—single chain; central—proto-fibril; bottom—super-fibril. Items K45 and E57 highlighted in the profile of a single chain—positions responsible for contact between proto-fibrils. Residues red—components of the hydrophobic core; Residues highlighted as yellow—the expected component of the hydrophobic core in a super-fibril, not present in the actual system.

A similar pattern is seen in the super-fibril structure in the amyloid available in PDB ID 7L7H. It shows the highest values of *RD* and *K* for super-fibril, while proto-fibril shows a micelle-like structure (Table 2, Figure 7).

The structure of a single chain reveals a system with a hydrophobic distribution consistent with the expected (Figure 7-upper). Distorting items in the structure with PDB ID 2N0A here matches their status to the *T* distribution as their location is surface—residues K45 and E57.

A micelle-like pattern is present for proto-fibril. The super-fibril contacts the single K45 and E46 residues on each chain. Such a system cannot be stabilized in the aquatic environment. Unfortunately, no publication is available describing this structure. The procedure of preparation is not publicly available. This is why it is difficult to recognize the experimental conditions which may possibly influence the structuralization of the super-fibril. The mechanism of the formation of this super-fibril is probably similar to the previously discussed example 6UFR (Figure 6).

**Figure 8:**
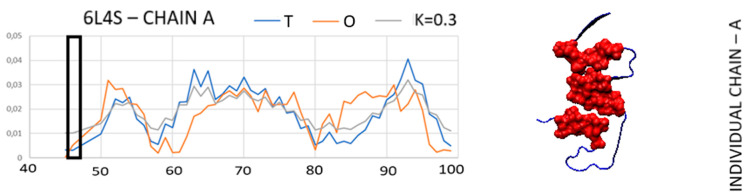

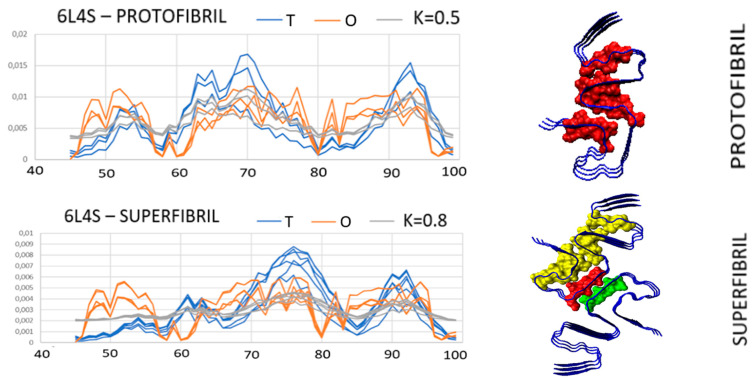
Summary of *T*, *O*, and *M* profiles for 6L4S: upper—single chain; central—proto-fibril; bottom—super-fibril. (two upper) Highlighted residues (red) show the location of the hydrophobic core. (bottom) yellow residues—the core in the proto-fibril, which in combination with the red and green fragments forms a common core for the super-fibril. The elimination of the remnants distinguished in the black box results in obtaining the value *RD* < 0.5.

Another example of a fibril with a relatively well-ordered hydrophobicity distribution is 6L4S (Figure 8). This amyloid represents several of the amyloids present here showing a slight breach of the *RD* = 0.5 threshold. This exceedance—similar to many globular proteins—is the result of a mismatch between $O_{i}$ and $T_{i}$ for individual residues and is therefore local in nature.

The reason for this elevated value of *RD* and *K* comes from individual residues exactly as discussed for A-Syn in the structure of 2N0A. The elimination of these residues from the calculation of the *RD* value reduces this value revealing a part of the system with a micelle-like distribution. Undoubtedly, however, environmental factors direct the structuring of the super-fibril based on polar interactions.

Despite the value of *RD* > 0.5, it is possible to identify individual residues disturbing the distribution of *O* locally in relation to the distribution *T* (Supplementary Materials, Tables S1–S3). Therefore, it is possible to determine the location of the hydrophobic core in the discussed examples of 6L4S amyloid structural units. In this case, the elimination of the three N-terminal residues (GVV) already results in *RD* < 0.5. Such a status for the N-terminal fragment may result from the dynamic freedom of the short chain segment exposed to the environment.

**Figure 9:**
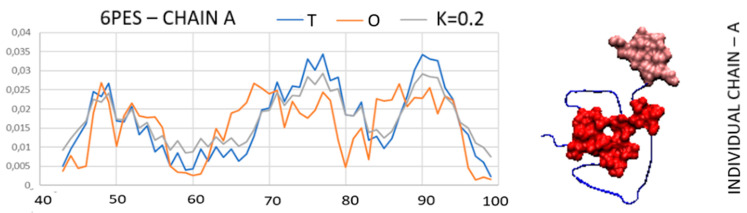

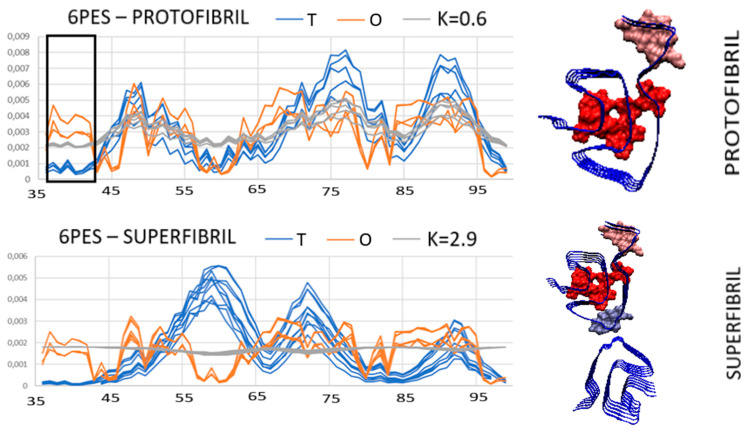
The *T*, *O*, and *M* profiles for: upper—a single chain devoid of the N-terminal segment showing a high match of the remaining part of the *O*-distribution chain with the *T* distribution. central—proto-fibril—N-terminal segment (black—on the profiles, pink—in the representation 3D) highlighted as increasing the *RD* value. bottom—super-fibril—pink segment—introducing a disturbance of the compliance of the *O* distribution against the *T* distribution. This section 55–65 in the profiles is represented as a significant deficit of hydrophobicity, where from the point of view of the micelle-like structure, maximum hydrophobicity is expected.

Another example is the amyloid code PDB ID 6PES. The identification of the hydrophobic core is simple here after the elimination of the N-terminal segment, which, by showing increased levels of hydrophobicity, disturbs the expected distribution. The summary of the *T*, *O*, and *M* distributions for the part of the chain devoid of the N-terminal segment (Figure 9.upper—segment distinguished as pink in the 3D representation) reveals a very high fit of the *O* and *T* distribution. Hydrophobicity on the surface is illustrated by the set of profiles in Figure 9 (the section is highlighted with a black frame).

Similar to the previous examples, stabilization of the super-fibril by a common hydrophobic core is not present here (ice-blue section—Figure 9, bottom—3D presentation).

The structure of the amyloid with PDB ID 6 PES (Fragment 36–94) is a similar example to that previously discussed. The slightly exceeded value of *RD* above 0.5 allows the identification of residues causing a mismatch of the *O* distribution with the *T* distribution. Here, the *O* distribution turns out to be very similar to the distribution present in 2NOA for the part 40–100. The composition of the core is comparable. The segment that overestimates the *RD* is segment 36–43. Its elimination from the calculations of *RD* and *K* results in a high fit of the distribution *O* to the distribution *T* (Supplementary Materials, Tables S1–S3). 

**Figure 10:**
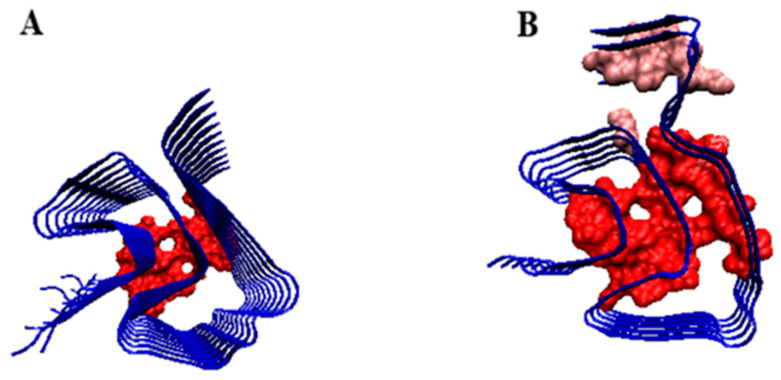
3D structure (**A**) fibrillar part 2N0A (red residues—hydrophobic core); (**B**) hydrophobic core system (red residues) in 6PES. Red residues—hydrophobic core; pink residues—residues of status incompatible with the micelle-like system showing a local excess of hydrophobicity.

The purpose of the visualization in Figure 10 is to show the high structural similarity of the compared fibrils. According to the interpretation based on the fuzzy oil drop model, the hydrophobic core system is dominant to the extent that it eliminates the share of the N-terminal segment (6PES) in the structure of the core as its component.

In this situation, it is surprising that a non-part of the chain mediated a super-fibril and is created. If the aquatic environment were to determine the formation of a super-fibril then the N-terminal part should be involved in the construction of the super-fibril. It, however, is formed via exposed polar residues. The marked ice-blue residues in the super-fibril (Figure 9—bottom line) show the area where the concentration of hydrophobic residues making up the interface is expected. However, this is not the case and the hydrophobicity deficit in the central part of the molecule, visible on the *T* and *O* profiles, represents the opposite state. Hence the extremely high value of K.

**Figure 11:**
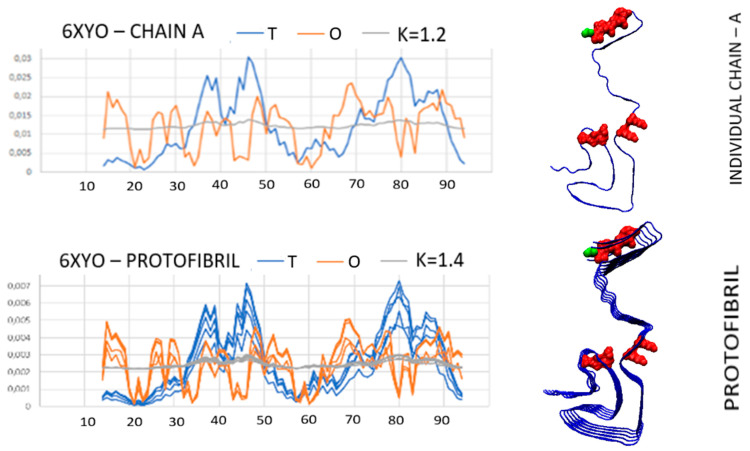

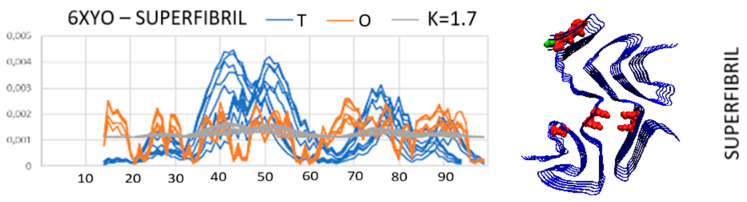
Summary of *T*, *O*, and *M* profiles for: upper—single chain; central—proto-fibril; bottom—super fibril brew with 3D structure visualization. The red residues introducing significant deviations from the *O* distribution to the *T* distribution are marked. These are the highly polar residues K34, K43, K45, and K80. The residue in green—N-terminal position for easy navigation.

An example of a highly disordered system is the distribution of *T*, *O*, and *M* present in amyloid with PDB ID 6XYO (Figure 11) This example of amyloid does not show micelle-like ordering for any of the forms discussed: single chain, proto-fibril, and super-fibril.

In the 6XYO amyloid structures, the positions of the polar residues are the main sources of mismatch of the *O* distribution with the *T* distribution. Similar to the previously discussed example, the N-terminal fragment showing local excess hydrophobicity and the polar residues (mainly Lys) in the central part of the chain cause a significant mismatch with the micelle-like system.

The analysis of the presented profiles reveals the importance of the positions of lysines: 34, 43, 45, and 80. These are the residues introducing a significant disturbance that exclude the presence of the micelle-like system. The listed amino acids (as shown by the profiles in Figure 11) introduce elements that are opposite to the expected system characteristic for the aquatic environment. High values of *K*, in all forms of *K* > 1.0 and in super-fibril significantly above 1.0, suggest that the folded protein recreates a completely different external force field. The influence of the aquatic environment is expressed as *K* = 0 or close to zero. Here, the *M* distribution can be treated as the outer field, which the folded protein has recreated by adapting the structure to such a system. The presence of the lysines mentioned in the areas where a high concentration of hydrophobicity is expected is significant. Thus, the effect of directing the folding process towards isolating the hydrophobic residues in the center and exposing the polar residues to the surface is not observed here.

This inconsistency with the fuzzy oil drop model is explained by the use of sarkosyl and SDS in the preparation of samples for the measurement of cryo-EM. The discussed amyloid (as well as in the amyloid with PDB ID 6XYP and 6XYQ) was obtained from biological material from patients diagnosed with multiple system atrophy (MSA), Parkinson’s disease, Parkinson’s disease dementia (PDD), and dementia with Lewy bodies (DLB). The *K* parameter expresses a measure of the degree of differentness of the environment from the aquatic environment, in particular the presence of hydrophobic factors. Therefore, high *K* values (comparable to those obtained for membrane proteins) may explain the specificity of the discussed system resulting from the specific environment which directs the location of hydrophobic residues on the surface (high *K* value). For this amyloid, identifying the part that meets the fuzzy oil drop model requires the elimination of a few residues to obtain an *RD* < 0.5. They are listed in Supplementary Materials, Tables S1–S3. They can be read from graphs where $O_{i}$ values do not match $T_{i}$ values or where $O_{i}$ significantly exceeds $T_{i}$ values. Of course, only those showing the greatest differences are eliminated. This procedure is performed stepwise until an *RD* < 0.5 is obtained.

**Figure 12:**
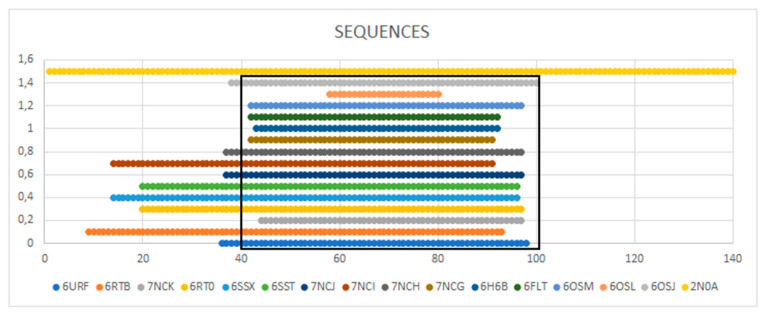
List of A-Syn chain fragments. Chain fragments representing micelle-like status are boxed.

The selected examples illustrate the diversity of the characteristics of the discussed amyloid forms of A-Syn. As it results from the given experimental conditions, the characteristics of the environment are of significant importance. The summary table (Figure 12) shows the location of that part of the A-Syn chain that tends to generate the amyloid form.

Both the *RD* and *K* values describing this example are comparable to the corresponding values obtained for water-soluble globular proteins. Enzymes are mainly in this group. In their case, as a rule, the residues disturbing the *O* system in relation to the *T* system are mainly catalytic residues or residues remaining in their immediate vicinity. Usually, it is a cavity that binds the substrate [46]. Similarly, a local excess of hydrophobicity on the surface may indicate the location of the interface in the case of the protein complex [47].

## 4. Discussion

In the discussion available in publications on obtaining amyloid forms of A-Syn in vitro, the presence of a lipid membrane forming a heterogenous lipid environment stimulating the formation of A-Syn amyloids is mentioned [48].

The presence of a heterogenous lipid environment inside mammalian cells has been associated with the formation of amyloid forms of A-Syn. Similarly, the presence of anionic lipids promotes different directions of aggregation [49]. Another example is the effect of polyphenols [50]. The polymorphic diversity of A-Syn amyloids was observed as dependent on ionic strength and protein concentration [51]. A supporting role towards the formation of an A-Syn amyloid is shown by the co-aggregation of A-Syn with S100A9 (calcium binding pro-inflammation protein) with polymorphic forms dependent on ionic strength [52,53]. Acceleration of the A-Syn amyloid transformation process was identified in the presence of zero-valent iron nanoparticles [54]. It has also been shown that the formation of A-Syn co-structures with lipids promotes the formation of amyloid fibrils [55]. The effect of pH change favoring the formation of the amyloid form of A-Syn was found at pH = 5.6 and 6.5, which was interpreted as a factor affecting the N-terminal chain fragment showing a significant presence of polar amino acids [56]. Insertion of certain fragments of oligomeric form of A-Syn to hydrophobic membrane seems to disturb the lipids and their packing and order, making the cellular membrane weaker, which was adopted as a contradiction to the A-Syn pore formation by the A-Syn oligomers [57,58,59]. Factors preventing the formation of amyloid fibrils were also determined. They include cinnamic acid derivatives [60]. 

The listed factors favoring or preventing the formation of A-Syn amyloids indicate a significant influence of the environment on the transformation process. It is therefore not surprising that the structural differentiation shown in the present work is. The differentiation is interpreted in the present work by the factor *K* in the target force field expression reproduced in the amyloid structure. Similarly—membrane proteins, depending on their function and diversified structure, reveal a different influence of the external factor (including the membrane in particular) on the shaping of the final structure of the protein ensuring the biological function—including channel formation in particular [13,14,15,61]. The *K* parameter introduced in the FOD-M model reveals the degree of alignment of the structure to the target distribution that is reproduced by the folded protein under the given conditions. Experimental studies also allowed to identify the sections: 1 (35–43) and (47–55), 2 (65–75), and (3) (83–90) as so-called hot positions [62]. The segment 35–43 in terms of the FOD-M model shows, in a large part of the fibrils discussed here, a significant mismatch in the hydrophobicity distribution with the micelle-like distribution. Segment 47–49 in the 6L4S also demonstrates this. Likewise, residues included in segment 83–90 exhibit this mismatch (e.g., 6L1T). It cannot be concluded, however, whether adjusting these segments would change the tendency towards amyloid transformation.

The solution, however—based on the FOD-M model—lies in the inability of A-Syn to form globular forms by the chain of this protein. In the conducted simulations of the folding of the A-Syn chain using the Robetta [63], I-TASSER [64], and UNRES [65] programs, they did not show a tendency to generate water-soluble globular forms. The simulations carried out by the UNRES package took into account the external force field in the form of additional (apart from non-binding interactions) optimization with respect to 3D Gauss of hydrophobicity ordering. Despite this operation, the A-Syn chain showed almost exclusively structures far from the forms with *RD* < 0.5. Similarly, the proposed A-Syn structure obtained with AlphaFold does not show a form with *RD* < 0.5 [66]. The structure predicted by this packet available in PDB ID A1Cb shows positions 1–90 in the form of α-helix and Random Coil structuring for the remainder of the polypeptide chain. This structure is close to that experimentally obtained in the micelle-bound form available in the PDB with ID 1XQ8. These structures do not show a globular form and do not show any similarity to amyloid forms at all.

The analysis of the WT structure and the amyloid A-Syn carried out in combination with the structural changes of another amyloid formed from the V L domain of the IgG chain and transthyretin reveals a different mechanism of amyloid transformation [15].

The structures of the analyzed proteins are available in both WT and amyloid form, which makes it possible to speculate about the transformation itself [15]. Comparing the status of these proteins indicates two distinct mechanisms of amyloid transformation. A-Syn under physiological conditions remains in contact with axon terminals of presynaptic neurons, which plays the role of a target maintaining the status of this protein very far from the globular form of water-soluble protein. Depriving A-Syn of this contact acting as a target introduces this protein into the water environment, where taking the form (revealed 2N0A) generates a structure characteristic of the aquatic environment. The form adapted to the aquatic environment is the amyloid form having an *RD* value < 0.5 and therefore, the form adapted to the polar external force field. Amyloid transformation of the V domain of light chain of IgG in laboratory conditions requires the use of factors changing the external force field for this protein. As a result, the WT protein with a status close to *RD* = 0.5 (the threshold value is slightly exceeded) in the changed environment obtains a structure with high *RD* values, which means a significant influence of the environment on the stabilization of the amyloid form of this protein [15].

In this light, the amyloid transformation of A-Syn is the result of the adaptation of the A-Syn chain to the aqueous environment, adopting fibril structuring consistent with the micelle-like system. This means that A-Syn released from the structuring target (high *K* for the WT form) to the low *K* form as a result of adaptation to the aquatic environment. External factors modifying the environment (including shaking as a factor introducing a significant increase in the presence of interphase (water–air) result in the adoption of an amyloid structure with a high *K* value [15].

The discussion on amyloid transformation includes topics related to the structuring of water, as such, the characteristics of which are variable, adapting to the presence of other factors and, consequently, influencing the processes taking place in its environment [67]. This mainly concerns the role of the presence of the air–water interphase, which in the case of amyloids is of particular importance due to the experimentally established ease of amyloid transformation by shaking the solution containing the protein. Shaking mainly increases the presence of the air–water interphase, which turns out to be a significant factor in the course of processes taking place in it [68,69,70].

The FOD-M model meets this issue by enabling the quantitative assessment of external factors in the form of the value of the *K* parameter.

The structural comparison of WT and the amyloid form of A-Syn is not possible due to the significant change in the significant share of the α-helix and the completely different shape of the tertiary structure, which makes the comparative analysis unfounded.

The influence of local conditions (used in experiments) is difficult to trace. The fibrils discussed in this paper were received using different procedures. Only a few of them are the products of long-lasting shaking (6OSM, 7NCK, 6PEO—6 weeks). Few examples are the results of patients’ tissue origin (6XYP, 6XYQ). Many of them are the results of a seeding procedure—addition of the fibrillar forms added to solutions of proteins expressed in bacteria—(6LRQ, 6A6B, 7NCJ). Few examples are mutants (7E0F, 6PES). In consequence, it is difficult to recognize the external conditions/structure relationship. The presented results may be treated as identification of the external force field differentiation expressed in quantitative form by the parameter K. The external conditions appear to influence the structuralization of particular fibrils. However, as it was shown in study [15], the A-Syn appears as an example of protein which, in natural conditions (physiological ones), represents the structure described by high K value. This high K value is the effect of a strong influence of environment (axon terminals of presynaptic neurons). The A-Syn chain losing contact with the natural target adopts the structure directed by the water environment. The examples discussed in this paper seem to prove this hypothesis. 

The structural analysis presented in this paper is aimed to make a link to the experimental and medical research of the pathology related to amyloidosis, especially to amyloidosis of A-Syn. The pathological effects of the altered forms of A-Syn are commonly called synucleinopathies. This altered form accumulates producing aggregates in neurons and glial cells. According to specific organization of these aggregates, the Parkinson’s disease, Lewy bodies, and multiple system atrophy are distinguished in medical treatment. Lewy bodies are characterized by spherical forms (in contrast to fibrilar forms in other synucleinopathies) localized in cytoplasm. They tend to move the position of organelles including the nucleus in the cell. The neuropathology of the A-Syn origin linked with Parkinson’s disease (PD) as well as Lewy bodies is identified by accumulation of misfolded forms of A-Syn which cause the devastating neurodegenerative disorders. One of the hypotheses of the origin of pathological consequences is the disorder in secretion of A-Syn causing the accumulation of prion-like forms [71,72]. This phenomenon is supported by the identified numerous single nucleotide polymorphisms of A-Syn which supports the fundamental changes from helical native form to β-structural in aggregated forms. The transfer of β-structural forms from affected cells to unaffected cells may serve as the seed for further propagation of large-scale fibrils causing neuronal dysfunction. The self-propagation of β-structural forms suggests the prion-like source of the neurodegeneration process [73]. The neurotransmitter deficiency is identified as a consequence of loss of mainly dopaminergic nerves. Particularly, the loss of neurons in the substantia nigra is observed in the final stage of Parkinson’s disease [74]. This observation supports the conclusions based on comparative analysis of WT structure (biologically active) demonstrating the conformation described by high values of RD and K. It means that the A-Syn in natural conditions keeps the biologically active form only for structures supported by the ‘permanent chaperon’ which is the complexation with neurons. Losing the contact with the target A-Syn adapts itself to water conditions, transforming its structure to water-directed formation of low RD and K values (the micelle-like organization represented in PDB ID—2N0A structural form [15]. 

The presence and influence of pathological forms of A-Syn identified on different cellular organelles including mitochondria. It is suggested also that the interaction of A-Syn with chromatin proteins undergoes the process of remodeling with special influence on chromatin marks and, in consequence, up-regulation of its own expression [75]. The therapeutic treatments originally focused on the application of high doses of D,L-DOPA [76]. Progress in therapy of neurodegenerative diseases of A-Syn origin is limited due to the lack of recognition of molecular mechanisms of this pathology [77,78,79]. This is why the search for molecular mechanisms of the process leading to pathological consequences is the focus of attention of many researchers [80]. 

This paper is proposing the molecular mechanism of amyloid transformation using the polymorphic structural forms of A-Syn as an example. Applying the structuralization criteria based on FOD and FOD-M models identifies the amyloid transformation of A-Syn as adopting the micelle-like structuralization after releasing the interaction with axon terminals of presynaptic neurons playing the role of a permanent chaperon stabilizing the structure highly different in respect to micelle-like organization.

## 5. Conclusions

The use of a modified FOD-M model taking into account the factors influencing protein folding other than water when applied to the forms of A-Syn amyloid seems to reflect the specificity of this process. The differentiation, both from the point of view of single-chain structuring, as well as proto-fibril and super-fibril, seems to be the result of adaptation to the characteristics of the environment. The presence of micelle-like amyloids as shown by A-Syn seems to be specific for this protein compared to other amyloids [71].

The concept proposed in study [15] in relation to A-Syn seems to be confirmed in the present analysis. It turns out that a very unusual form A-Syn shows in its native form (*RD* = 0.643, *K* = 1.3) results from its interaction with the target, which is axon terminals of presynaptic neurons which determines this state; after the release of this significant factor, the A-Syn chain behaves typically for proteins, i.e., it restores the micelle-like structure being only under the influence of the water environment. Factors present in the conditions of obtaining the forms available for cryo-EM research influence the differentiation of the structural forms of A-Syn amyloids. However, this variation is not as significant as it is in the case of transmembrane proteins associated with the refined function of ion channels.

The presence and interpretation of the *K* parameter in the FOD-M model expressing the influence of factors other than water on the structuring of proteins, including amyloid proteins, in particular, is closely related to experimental observations showing a significant undergoing to external factors of A-Syn structuring [81,82].

The use of the FOD-M model taking into account the presence of other than the polar water environment leads to an answer to the question of structuring under the influence of external factors. The specificity of amyloid fibril structures also lies in the specific network of hydrogen bonds. A form of recording of the external field influencing and favoring the construction of the hydrogen bond network is being sought.

It should be noted that the proposed model for A-Syn amyloid transformation cannot be generalized to other known examples of amyloid proteins, as demonstrated in study [15].

The different values of the *RD* and *K* parameters for the proteins in question (including the amyloid forms A-Syn) are the basis for the simulation of protein folding processes in this process leading to the formation of amyloid forms (in particular A-Syn). Accounting for the presence of water in the form of a set of single H_2_O molecules and including them in the form of non-binding interactions turns out to be insufficient. A mathematical description of the form of a varied external force field in the form of a continuum influencing the protein folding process is planned as a component of the force field in protein folding programs, where it would be possible to take into account the diverse form of the environment actively involved in the folding process, including misfolded forms [71,72].

The model presented can be applied in any protein for any issue characterizing the protein structure. The phenomena of water influence on folding as well as the influence on other compounds (hydrophobic compounds in particular) are universal and so is the applied model. 

## Figures and Tables

**Table 1:** List of proteins (chain selected and its fragment) analyzed in this paper. The protein given in bold—reference protein with complete length of the chain as well as its fragment selected for detailed analysis—and fragment participating in fibril formation.

PDB ID	Chain	Fragment	Ref.
6CU8	E	43–83	[17]
6CU7	D	43–83	[17]
6L1T	C	1–100	[17]
6RTB	C	36–98	[18]
6SST	C	14–96	[18]
6SSX	C	14–97	[18]
6RT0	A	37–97	[18]
6UFR	A	36–99	[19]
7L7H	B	61–98	[20]
7NC1	C	37–97	[21]
7NCJ	C	14–96	[21]
7NCA	D	37–97	[21]
7NCG	A	14–91	[21]
7NCH	C	14–91	[21]
7NCK	C	9–93	[21]
7LC9	A	46–96	[22]
7LC9	C	46–98	[22]
6A6B	C	38–97	[23]
6L4S	A	46–96	[24]
6XYO	B	21–99	[25]
6XYP	B	36–99	[25]
6XYP	A	14–94	[25]
6XYO	A	14–94	[25]
6H6B	E	37–97	[26]
6FLT	E	38–95	[26]
6OSL	E	37–96	[27]
6OSJ	E	39–97	[27]
6OSM	E	38–95	[27]
7E0F	B	37–99	[28]
6LRQ	B	37–99	[29]
6PEO	A	36–99	[30]
6PES	A	14–94	[30]
6XYQ	B	36–99	[31]
6XYQ	A	14–94	[31]
7C1D	A	37–97	[32]
**2N0A**	**B**	**1–140**	[33]
	**B**	**38–100**	

**Table 2:** Summary of parameters (fuzzy oil drop model) *RD* and *K* for A-Syn amyloids available from PDB. Parameters were determined for structural units: single chain, proto-fibril, and super-fibril—if present.. The 2N0A structure is taken as a reference as it only represents the complete A-Syn chain. List of proteins ordered by increasing *RD* value. Proteins distinguished as bold—the detailed analysis of these proteins is presented in the Results.

			Chain		Proto-Fibrils		Super-Fibrils	
PDB ID	Chains	Fragment	*RD*	*K*	*RD*	*K*	*RD*	*K*
**6CU8**	E	43–83	0.384	0.2	0.458	0.3	0.437	0.3
6RTB	C	36–98	0.438	0.3	0.489	0.3	0.738	2.1
**6UFR**	A	36–99	0.453	0.3	0.453	0.3	0.673	1.1
**7L7H**	B	61–98	0.463	0.3	0.495	0.4	0.808	4.4
7NCI	C	37–97	0.468	0.3	0.452	0.3	0.744	1.8
6SST	C	14–96	0.484	0.3	0.508	0.4	0.757	2.0
7LC9	A	46–96	0.487	0.3	0.490	0.3		
6SSX	C	14–97	0.490	0.3	0.496	0.4	0.689	1.3
7NCJ	C	14–96	0.496	0.4	0.461	0.3	0.715	1.6
6RT0	A	37–97	0.501	0.4	0.506	0.4	0.910	1.3
7NCA	D	37–97	0.502	0.4	0.481	0.4	0.703	1.3
6A6B	C	38–97	0.511	0.3	0.538	0.4	0.663	0.9
7NCG	A	14–91	0.516	0.4	0.483	0.4	0.758	1.9
**6L4S**	A	46–96	0.517	0.4	0.547	0.5	0.620	0.7
7NCH	C	14–91	0.518	0.4	0.472	0.4	0.672	1.1
6XYO	B	21–99	0.526	0.4	0.541	0.5		
6H6B	E	37–97	0.540	0.4	0.572	0.5	0.750	1.6
6OSL	E	37–96	0.540	0.4	0.549	0.4	0.697	1.1
6OSJ	E	39–97	0.547	0.4	0.570	0.5	0.704	1.2
7E0F	B	37–99	0.551	0.6	0.544	0.5	0.633	0.8
6LRQ	B	37–99	0.553	0.4	0.571	0.5	0.765	2.9
6FLT	E	38–95	0.565	0.4	0.600	0.6	0.770	1.8
6XYP	B	36–99	0.569	0.5	0.581	0.5		
6PEO	A	36–99	0.581	0.4	0.581	0.6		
6XYQ	B	36–99	0.584	0.4	0.585	0.5		
**6PES**	A	14–94	0.584	0.5	0.582	0.6	0.769	2.9
7NCK	C	9–93	0.585	0.4	0.548	0.5		
6CU7	D	43–83	0.599	0.5	0.663	1.0	0.706	1.3
6OSM	E	38–95	0.606	0.4	0.607	0.5	0.731	1.4
6L1T	C	1–100	0.650	1.0	0.657	1.2	0.776	2.2
7C1D	A	37–97	0.702	0.7	0.653	0.8	0.746	1.2
7LC9	C	46–98	0.710	1.1	0.669	1.1	0.660	1.1
6XYP	A	14–94	0.719	1.0	0.704	1.3	0.729	1.6
**6XYO**	A	14–94	0.720	1.2	0.706	1.4	0.711	1.7
6XYQ	A	14–94	0.721	1.0	0.707	1.3	0.731	1.6
**2N0A**	B	1–140	0.718	1.4	0.472	0.3		
	B	38–100	0.495	0.3	0.492	0.3		

## Data Availability

All data are available upon request addressed to the corresponding author. The program allowing calculation of *RD* is accessible on the GitHub platform: https://github.com/KatarzynaStapor/ FODmodel and on the platform https://hphob.sano.science (accessed on 14 January 2023).

## Supplementary Materials

The following supporting information can be downloaded at: https://www.mdpi.com/article/10.3390/biomedicines11051324/s1, Table S1: RD i K values for individual chains; Table S2: Values of RD i K as calculated for proto-fibrils; Table S3: Values of RD and K for super-fibrils.
