# Supplementary material for: Structural Specificity of Polymorphic Forms of α-Synuclein Amyloid
Source: Biomedicines. 2023 Apr 29;11(5):1324. doi: 10.3390/biomedicines11051324 (PMC10216396; doi:10.3390/biomedicines11051324)
Supplement: Supplementary file 1 [file biomedicines-11-01324-s001.zip › biomedicines-2313153-supplementary.pdf]

SUPPLEMANTARY MATERIALS

Table S1. RD i K values for individual chains. To define the part od the structuralisation micelle-like some residues (of high difference between Ti and Oi) got eliminated from Ti and Oi calculation. Dispersed – residues of high differences between Ti and Oi dispersed all over the chain – the procedure of elimination does not solve the problem. Highly dispersed – examplex of high degree of disorder (FOD-model criteria). The order of proteins – as in Table 1.

			Individual chain		Residues eliminated		
PDB ID	CHAINS	FRAGMENT	RD	K	RD	K	FRAGMENT
6CU8	E	43-83	0.384	0.2			
6RTB	C	36-98	0.438	0.3			
6UFR	A	36-99	0.453	0.3			
7L7H	B	61-98	0.463	0.3			
7NCI	C	37-97	0.468	0.3			
6SST	C	14-96	0.484	0.3			
7LC9	I	46-96	0.487	0.3			
6SSX	C	14-97	0.490	0.3			
7NCJ	C	14-96	0.496	0.4			
6RT0	A	37-97	0.501	0.4			
7NCA	D	37-97	0.502	0.4			
6A6B	C	38-97	0.511	0.3	0.370	0.0	37-42
7NCG	A	14-91	0.516	0.4	0.473	0.3	14-21
6L4S	A	46-96	0.517	0.4	0.471	0.3	47-49
7NCH	C	14-91	0.518	0.4	0.473	0.3	14-20
6XYO	B	21-99	0.526	0.4	0.474	0.3	43-46
6H6B	E	37-97	0.540	0.4	0.490	0.3	44-94
6OSL	E	37-96	0.540	0.4	0.435	0.2	N-TERMINAL
6OSJ	E	39-97	0.547	0.4	0.486	0.3	N-TERMINAL
7E0F	B	37-99	0.551	0.6	0.485	0.7	DISPERSED
6LRQ	B	37-99	0.553	0.4	0.435	0.2	43-99
6FLT	E	38-95	0.565	0.4	0.491	0.3	43-93
6XYP	B	36-99	0.569	0.5	0.410	0.1	36-42
6PEO	A	36-99	0.581	0.4	0.422	0.2	38-42
6XYQ	B	36-99	0.584	0.4	0.419	0.1	36-42
6PES	A	14-94	0.584	0.5	0.425	0.2	36-43
7NCK	C	9-93	0.585	0.4	0.416	0.4	38-42
6CU7	D	43-83	0.599	0.5	0.497	0.3	38-42
6OSM	E	38-95	0.606	0.4	0.432	0.2	N-TERMINAL
6L1T	C	1-100	0.650	1.0			HIGHLY DISPERSED
7C1D	A	37-97	0.702	0.7	0.455	0.2	N-TERMINAL
7LC9	C	46-98	0.710	1.1	0.497	0.4	DISPERSED
6XYP	A	14-94	0.719	1.0	0.491	0.3	14-20,25-31,39,43-46,79,80,93,94
6XYO	A	14-94	0.720	1.2			HIGHLY DISPERSED
6XYQ	A	14-94	0.721	1.0	0.497	0.3	12-19+DISPERSED
2N0A	B	1-140	0.718	1.4	0.495	0.4	35-99

Table S2. Values of RD i K as calculated for proto-fibrils. The values given in bold – the nmicelle-like construction (RD<0.5). The right column – the fragment truncated. No – denotes absence of following residues.

			Proto-fibril		Residues eliminated		
**PDB ID**	**CHAINS**	**FRAGMENT**	**RD**	**K**	**RD**	**K**	**Fragment No - eliminated**
6CU8	A-I	43-83	0.458	0.3			
6RTB	A-E	9-93	0.489	0.3			
6URF	ADEHI	36-98	0.453	0.3			
7L7H	A-D	36-79	0.495	0.4			
7NCI	A-F	14-91	0.452	0.3			
6SST	A-E	14-96	0.508	0.4	0.489	0.4	20-96
7LC9	ABCDEG	46-96	0.669	1.1	0.474	0.4	58,61,65-69,75,78,80
6SSX	A-E	14-96	0.496	0.4			
7NCJ	A-F	37-97	0.461	0.3			37-97
6RT0	A-E	14-97	0.506	0.4	0.493	0.4	20-97
7NCA	AEFJKL	37-97	0.481	0.4			
6A6B	A-F	37-99	0.538	0.4	0.392	0.1	44-99
7NCG	AEFJKL	37-97	0.483	0.4			37-97
6L4S	ACE	45-99	0.547	0.5	0.499	0.4	20-24,43,51-56,32-35,38-40,84-88
7NCH	A-F	14-91	0.472	0.4			
6XYO	BDFHJ	21-99	0.541	0.5	0.497	0.4	21-99 NO 43-46,80
6H6B	A-H	38-95	0.572	0.5	0.472	0.3	42-91
6OSL	ACEGI	39-97	0.549	0.4	0.428	0.2	42-97
6OSJ	ACEGI	37-97	0.570	0.5	0.480	0.3	43-97
7E0F	A-C	50-98	0.544	0.5	0.496	0.4	52-57,77,82
6LRQ	A-C	37-99	0.571	0.5	0.454	0.3	43-99
6FLT	A-I	38-95	0.600	0.6	0.482	0.3	43-92
6XYP	BDFHJ	36-99	0.581	0.5	0.425	0.3	43-99
6PEO	A-E	36-99	0.581	0.6	0.425	0,1	43-99 NO 45,46,50,80
6XYQ	BDFHJ	36-99	0.585	0.5	0.410	0.1	43-99
6PES	A-E	36-99	0.582	0.6	0.430	0.2	43-94
7NCK	A-F	37-97	0.548	0.5	0.414	0.2	44-97
6CU7	A-I	38-97	0.663	1.0	0.499	0.4	38-41,45,46,58,66-69,81,82,84-86,9394
6OSM	ACEGI	37-96	0.607	0.5	0.416	0.2	42-92
6L1T	A-E	1-100	0.657	1.2	0.462	0.3	
7C1D	ABE	46-96	0.653	0.8	0.403	0.3	50-80,83-99
7LC9	FHIJK	61-98	0.490	0.3			
6XYP	ACGEI	14-94	0.704	1.3	0.493	0.4	32-90 NO 43-46,34,35,78-80,54-56
6XYO	ACEGI	14-94	0.706	1.4	0.491	0.3	21-93 NO 24-27, 32-35, 43to46. 51-56. 79,80,83
6XYQ	ACEGI	14-94	0.707	1.3	0.498	0.3	32-90 NO 43-46 and 52-55
2N0A		1-140	0.472	0.3	0.473	0.4	1-42, 101-140*
2N0A		38-100	0.492	0.3			

Table S3. Values of RD and K for super-fibrils. The order of proteins as in Table 1.

		Super-fibril		Truncated Super-fibril		
**PDB ID**	**Fragment**	**RD**	**K**	**RD**	**K**	**Fragment eliminated**
6CU8	43-83	0.437	0.3			
6RTB	36-98	0.738	2.1			
6UFR	36-99	0.673	1.1	0.423	0.2	
7L7H	36-79	0.808	4.4			
7NCI	37-97	0.744	1.8	0.483	0.4	No 14-20,42-46,50,81-97
6SST	14-96	0.757	2.0			
7LC9	50-98/ 46-96	0.660	1.1			Dispersed
6SSX	14-97	0.689	1.3	0.490	0.3	No 26-35,42-48,50,51,60,62-70,80,82,84-97
7NCJ	14-96	0.715	1.6	0.475	0.4	14-19,43-46,81-97
6RT0	37-97	0.691	1.3	0.480	0.3	No14-20,36-40,84-97
7NCA	37-97	0.703	1.3	0.490		No 37-43,81-95
6A6B	38-97	0.663	0.9	0.493	0.4	37-42,66-71,93,94
7NCG	14-91	0.758	1.9	0.492	0.5	No 42-46,81-97
6L4S	46-96	0.620	0.7	0.433	0.3	45-58,60,61, 75,79,80,84-86
7NCH	14-91	0.672	1.1	0.498	0.3	No14-19,37,4081,82,84-91
6XYO	14-94	0.711	1.7	0.495	0	No 42-46, 63-71,50,95
6H6B	37-97	0.750	1.6	0.467	0.2	No 86-96 37-44,67-70,81-84
6OSL	37-96	0.697	1.1	0.481	0.3	No 37-41,65-70,63,75,81,84,85,91
6OSJ	39-97	0.704	1.2	0.494	0.4	No 37-41, 92-97,65-69, 53,54,57,58,75,91
7E0F	37-99	0.633	0.8	0.497	0.4	50-57,68,75, 79,80,82,92
6LRQ	37-99	0.765	2.9			
6FLT	38-95	0.770	1.8	0.431	0.2	No 38-42, 63-71, 81-94
6XYP	14-94	0.729	1.6	0.487	0.4	No 14-33,43-46,50,65-70,
6XYQ	14-94/36-99	0.731	1.6	0.499	0.4	No 29-31,35,38-46,50,65-67,70,80,89-C
6PES	14-94	0.769	2.9			
6CU7	43-97	0.706	1.3	0.434	0.2	43- 95 No 64-70, 81,82,84,86,91-95
6OSM	38-95	0.731	1.4	0.478	0.3	No 37-42,50,57,58,66-69,82,85,88-96
6L1T	1-100	0.776	2.2			
7C1D	37-97	0.746	1.2	0.484	0.4	No 46-58,79,80,84-87
